# Prevalence of Online Sexual Offenses Against Children in the US

**DOI:** 10.1001/jamanetworkopen.2022.34471

**Published:** 2022-10-14

**Authors:** David Finkelhor, Heather Turner, Deirdre Colburn

**Affiliations:** 1 Crimes Against Children Research Center, University of New Hampshire, Durham

## Abstract

**Importance:**

Sexual abuse is increasingly facilitated by technology, but the prevalence and dynamics of such offenses have not been well delineated, making it difficult to design prevention strategies.

**Objective:**

To examine the frequency and characteristics of online and technology-facilitated sexual abuse against children and youth.

**Design, Setting, and Participants:**

In this nationally representative online survey study performed from November 19 to December 29, 2021, young adults aged 18 to 28 years were asked retrospectively about their childhood (<18 years) experiences of online and technology-facilitated abuse. The 2639 participants were sampled from an online panel.

**Main Outcomes and Measures:**

Participants were asked questions about 11 different kinds of online and technology-facilitated sexual abuse with follow-up questions about their dynamics and offenders. Prevalence rates were calculated for several cross-cutting concepts (online child sexual abuse, image-based sexual abuse, self-produced child sexual abuse images, nonconsensual sexting, online grooming by adults, revenge pornography, sextortion, and online commercial sexual exploitation). Survey weights were applied to obtain population prevalence estimates.

**Results:**

A total of 2639 individuals (48.5% male, 49.8% female, and 1.8% other gender; 23.7% Hispanic, 12.6% non-Hispanic Black, 53.9% non-Hispanic White, 4.8% other race, and 5.0% ≥2 races) were surveyed. Childhood (before 18 years of age) prevalence rates were as follows: online child sexual abuse, 15.6% (SE, 1.0%); image-based sexual abuse, 11.0% (SE, 0.9%); self-produced child sexual abuse images, 7.2% (SE, 0.7%); nonconsensual sexting, 7.2% (SE, 0.7%); online grooming by adults, 5.4% (SE, 0.5%); revenge pornography, 3.1% (SE, 0.5%); sextortion, 3.5% (SE, 0.6%); and online commercial sexual exploitation, 1.7% (SE, 0.3%). The prime age of vulnerability across all categories was 13 to 17 years. Perpetrators in most categories were predominantly dating partners, friends, and acquaintances, not online strangers.

**Conclusions and Relevance:**

The results of this national survey study suggest that a considerable portion of youth have experienced online child sexual abuse. Professionals planning prevention and intervention strategies for online sexual abuse should understand that dynamics include diverse episodes that are often extensions of dating abuse, sexual bullying, and sexual harassment, not only events perpetrated by adult internet predators.

## Key Points

**Question:**

What are the lifetime prevalence and characteristics of online and technology-facilitated sexual abuse against children and youth?

**Findings:**

In this national survey study of 2639 individuals, lifetime exposures were as follows: online child sexual abuse, 15.6%; image-based sexual abuse, 11.0%; self-produced child sexual abuse images, 7.2%; nonconsensual sexting, 7.2%; online grooming by adults, 5.4%; revenge pornography, 3.1%; sextortion, 3.5%; and online commercial sexual exploitation, 1.7%.

**Meaning:**

Varied subtypes of online sexual abuse have different prevalence rates.

## Teaser

This survey study examines the frequency and characteristics of online and technology-facilitated sexual abuse against children and youth in the US.

## Introduction

Technology has created new modalities for the sexual abuse and exploitation of children. Adults use social media to target children for illegal sexual activities. Dating partners and peers use digital devices to take and misuse sexual images of their friends. Many variations of these abuse dynamics have been identified by educators,^1^ physicians,^2,3^ police,^4^ and parents and have been labeled with names such as online sexual abuse,^5^ online sexual solicitation,^6^ online grooming,^7^ image-based sexual abuse,^8^ child sexual abuse image production,^9^ sextortion,^10,11^ and nonconsensual sexting.^12^

These new offenses have disrupted traditional understandings of child sexual abuse—sexual activity with a child that is nonconsensual because of it being coerced, being unwanted, or involving an impermissible age difference. The variety of technology-facilitated modalities may have different dynamics and risk factors that imply the need for new kinds of prevention and intervention strategies. Although this area of research has begun to attract more interest,^13,14^ much more empirical work is needed.

One need is to better describe the scope and diversity of these offenses that have been labeled with an array of different terms. The popular narrative of adult internet predators is often reflexively applied to these offenses by journalists and advocates, yet even studies that focus exclusively on police arrest cases show a great diversity of offenses, including events frequently perpetrated by other youth, friends, family, and romantic partners.^15^ To compound the problem, cases with these latter dynamics may often be underreported in data from police or child protection agencies. The current study used a nationally representative population survey to categorize and define many of these new technological modalities of abuse, estimate their prevalence, and demonstrate how they can be assembled from a single survey into the various conceptual categories that have become part of the research and policy conversation.

## Methods

This survey study was conducted from November 19 to December 29, 2021, using a nationally representative online panel (KnowledgePanel, Ipsos). KnowledgePanel members were recruited by random probability sampling from mail addresses gleaned from national universal address databases. Digital devices were provided to any recruited sample members who lacked devices to participate. All panelists aged 18 to 28 years (n = 13 884) were solicited online (no in-person contact) for the current survey. All panelists responded to a consent statement that preceded the questionnaire. In total, 2639 panel members participated in the survey by the end of data collection, with an overall study completion rate of 20% and a cumulative response rate of 1%, taking into account the original panel recruitment statistics.^16^ Such response rates are not untypical of modern survey research, and the KnowledgePanel design has been shown to be on par with what more traditional survey methods can currently provide.^17,18,19^ The study was approved and overseen by the University of New Hampshire Human Subjects Review Board. This study followed the American Association for Public Opinion Research (AAPOR) reporting guideline.

Of the 2639 participants who completed surveys, 933 reported 1 or more retrospective episodes (before 18 years of age) in response to screening questions about technology-facilitated abuse. For those with multiple episodes, the survey gathered follow-up information on 2 episodes, prioritizing episodes that were at the youngest age and of less frequent occurrence in the sample overall, as determined by a survey pretest. The final recruited sample was slightly older and more often female compared with the US population distribution of individuals aged 18 to 28 years. Weights were developed for the sample that adjust for nonresponse (raking adjustment to the 2021 age-specific benchmarks for gender, educational level, race and ethnicity, household income, US Census region, and metropolitan status) and the prioritization of lower base-rate incidents among those with multiple exposures.

The study operationalized several distinct offenses that occur under broader concepts from the literature,^5,8,14^ such as image-based sexual abuse, online grooming, and nonconsensual sexting. Outlined below are 11 screening questions with yes/no responses that, in various combinations, operationalized these broader categories of offenses. These questions were developed from a review of previous questionnaires and consultation with experts in the field.

### Nonconsensual Image Misuse

“Has someone ever shared with other people a sexual picture or video of you without your permission?” This question was meant to include episodes in which someone may have had consensually obtained an image but then shared it with others for whom it was not intended. This could include the sharing of an image that was not initially obtained consensually.

### Nonconsensual Image Taking

“Has someone ever taken or made a sexual picture or video of you without your permission?” This question was meant to include images of the child being abused or having sexual images taken when the child was unconscious, intoxicated, distracted, or unable to consent. It could also include so-called deepfake images in which a child’s head or likeness was imposed on a sexual image of someone else.

### Forced Image Recruitment

“Has someone ever threatened, tried to force you, or strongly pressured you to provide sexual pictures or videos online or through a cell phone?” This question was meant to include episodes of someone trying to coerce images when the target was unwilling or reluctant. It could also include a romantic partner pressuring or badgering the individual into providing an image. An image need not to have been provided.

### Threatened Sharing

“Has someone ever threatened to share a sexual picture or video of you to get you to do something—like take or send other sexual pictures of yourself, have a sexual relationship with them, pay them money, or something else?” This could included episodes in which a perpetrator claimed to be in possession of sexual images and was threatening to misuse them unless the target did something for them.

### Unwanted Sexual Talk

“Did anyone ever use the internet or a cell phone to try to get you to talk about sex when you did not want to?” This category could include very brief or casual inquiries.

### Unwanted Sexual Questions

“Did anyone ever use the internet or a cell phone to ask you for sexual information about yourself when you did not want to answer those questions? This means very personal questions, like what your body looks like or sexual things you have done?” As with unwanted sexual talk, this category could include very brief or casual inquiries.

### Unwanted Sexual Acts Requests

“Did anyone ever use the internet or a cell phone to ask you to do something sexual that you did not want to do?” As with unwanted sexual talk and unwanted sexual questions, this category could include very brief or casual inquiries.

### Voluntary Older Partner

“Did you have intimate sexual conversations or share sexual pictures or videos (online or through a cell phone), even if you wanted to, with a person who was 5 or more years older than you?” This question was meant to capture voluntary sexual interactions with an older partner.

### Commercial Talk, Images, or Other Sexual Activity

“Have you done any of the following things over the internet or a cell phone (including texting) in exchange for money, drugs, or other valuable items?” Participants provided yes/no responses to 3 sources of commercial exploitation: sexual talk; making, sending, or posting sexual pictures or videos of oneself; or any other sexual activity. This question was meant to capture incidences of youths using technology to earn money or get valuables by providing sexual services.

We grouped the prevalence rates associated with these individual screener questions (or portions of them) into aggregates that represented broader concepts that have been articulated in the literature^20^ (Table 1). The following categories describe the aggregates.

**Table 1.** Prevalence Rate for Each Screener and Composite Offense Category

Offense	No. of participants (N = 2639)	Item prevalence	Online solicitation (n = 732)	Online child sexual abuse (n = 494)	Image-based sexual abuse (n = 335)	Nonconsensual sexting (n = 222)	Self-produced child sexual abuse images (n = 234)	Online grooming with adult perpetrator (n = 208)	Revenge pornography (n = 80)	Sextortion (n = 81)	Online commercial exploitation (n = 58)
Weighted offense prevalence (SE), %	NA	NA	22.5 (1.2)	15.6 (1.0)	11.0 (0.9)	7.2 (0.7)	7.2 (0.7)	5.4 (0.5)	3.1 (0.5)	3.5 (0.6)	1.7 (0.3)
Components of offense category, mean (SE), %											
Nonconsensual image sharing	131	4.9 (0.6)	NA	4.9 (0.6)	4.9 (0.6)	4.9 (0.6)	4.9 (0.6)	NA	2.7 (0.5)^a^	NA	NA
Nonconsensual image taking	62	2.0 (0.3)	NA	2.0 (0.3)	2.0 (0.3)	2.0 (0.3)	NA	NA	0.7 (0.2)^a^	NA	NA
Forced image	353	10.3 (0.8)	NA	1.6 (0.3)^b^	1.6 (0.3)^b^	1.6 (0.3)^b^	1.6 (0.3)^b^	1.1 (0.2)^c^	NA	NA	NA
Threatened sharing	81	3.5 (0.6)	NA	3.5 (0.6)	3.5 (0.6)	NA	NA	NA	NA	3.5 (0.6)	NA
Unwanted sexual talk	568	16.9 (1.0)	16.9 (1.0)	1.2 (0.2)^c^	NA	NA	NA	1.2 (0.2)^c^	NA	NA	NA
Unwanted sexual questions	621	18.8 (1.1)	18.8 (1.1)	1.5 (0.3)^c^	NA	NA	NA	1.5 (0.3)^c^	NA	NA	NA
Unwanted sexual act requests	482	14.3 (1.0)	14.3 (1.0)	1.1 (0.3)^c^	NA	NA	NA	1.1 (0.3)^c^	NA	NA	NA
Voluntary older partner	285	8.6 (0.7)	NA	8.6 (0.7)	3.1 (0.5)^d^	NA	3.1 (0.5)^d^	2.7 (0.4)^c^	NA	NA	NA
Commercial talk	49	1.5 (0.3)	NA	1.5 (0.3)	NA	NA	NA	0.3 (0.1)^c^	NA	NA	1.5 (0.3)
Commercial images	38	1.1 (0.2)	NA	1.1 (0.3)	1.1 (0.3)	NA	1.1 (0.3)	0.3 (0.0)^c^	NA	NA	1.1 (0.3)
Commercial other	30	0.8 (0.2)	NA	0.8 (0.2)	NA	NA	NA	0.4 (0.1)^c^	NA	NA	0.8 (0.2)

Abbreviation: NA, not applicable.

^a^ With intent to harm.

^b^ With image provided.

^c^ With adult perpetrator only.

^d^ Image-based interaction only.

### Online Solicitation 

Online solicitation included unwanted solicitations (unwanted sexual talk, unwanted sexual questions, and unwanted sexual act requests) by adults or other youth (known or unknown). Because so many of these episodes were brief and of unknown source, only those from known adults are included in subsequent categories.

### Online Child Sexual Abuse

Online child sexual abuse, also called online child sexual exploitation and abuse, included all the individual screener questions, even those that had no image content. For the unwanted solicitation items, only solicitations by adults were counted as qualifying as sexual abuse.^21^

### Image-Based Sexual Abuse 

Image-based sexual abuse included episodes from all the individual screener questions that applied to images—misuse of images, threats or pressure to obtain images, voluntarily provided images in a statutorily impermissible relationship, and the provision of images for commercial purposes.^8^ For the forced image category and for voluntary older partner, only episodes in which images were actually provided were counted.^6,13,22^

### Nonconsensual Sexting

The nonconsensual sexting aggregate combined both nonconsensual sharing of images and the nonconsensual taking or production of images. The portion of forced image episodes that resulted in an image being provided was counted as well.

### Self-produced Child Sexual Abuse Images

Self-produced child sexual abuse images included youth providing their own images to perpetrators who nonconsensually shared them. This type of abuse also included forced image episodes in which an image was shared, as well as the voluntary sharing of images with adults or in commercial image transactions. This type of abuse does not include voluntary image sharing with a peer.

### Online Grooming by an Adult 

Online grooming by an adult included unwanted solicitation and forced images but only when they involved presumed or known adults.^7,23^ This type of abuse also included voluntary older partner contact and commercial sexual exchange activities (commercial talk, images, or other) when adults were involved.

### Revenge Pornography

Revenge pornography included sexual images that had been provided or taken without consent (nonconsensual sharing and nonconsensual image taking) but only where follow-up questions determined that the misuse was to intentionally hurt or humiliate the target.^24^ Other motives for nonconsensual use, such as showing off, were not counted.

### Sextortion or Sexual Extortion of Children

The term *sextortion* has been generally used to describe someone making a threat to disseminate sexual images in their possession to obtain money, additional pictures, or other sexual activities.^11,20^ Sextortion is equivalent to the single screener question on threatened sharing.

### Online Commercial Exploitation

Online commercial exploitation aggregated the 3 types of providing sexual services for reward. These included commercial talk, commercial images, or other commercial sexual activity.

### Statistical Analysis

Data were analyzed using Stata/SE software, version 17 (StataCorp LLC). Survey weights were applied during analyses to obtain population prevalence estimates. Only episodes occurring before 18 years of age were counted. Episodes in adulthood were available from the survey but were excluded in this analysis. To determine whether there were significant differences between child or perpetrator characteristics, we conducted Pearson χ^2^ tests. A 2-sided *P* < .05 was considered to be statistically significant.

## Results

The sample consisted of 2639 individuals (48.5% male, 49.8% female, and 1.8% other gender [trans male, trans female, or gender fluid/nonconforming]; 23.7% Hispanic, 12.6% non-Hispanic Black, 53.9% non-Hispanic White, 4.8% other race [option on the survey], and 5.0% of ≥2 races). The most frequently reported types of screener episodes (with their prevalence rates) were unwanted sexual questions that occurred online (18.8% [SE, 1.1%]), unwanted sexual talk (16.9% [SE, 1.0%]), and unwanted requests to engage in sexual acts (14.3% [SE, 1.0%]) (Table 1). Aggregated together as online solicitation, these episodes were encountered during childhood by 22.5% (SE, 1.2%) of the sample. This aggregated category included a large proportion of episodes (74.8% [SE, 2.7%]) that were brief enough that targets did not know much about the solicitor (Table 2).

**Table 2.** Child and Perpetrator Age and Relationship for Each Offense Category

Offense	Weighted prevalence rate (SE), %^a^								
	Online solicitation (n = 732)	Online child sexual abuse (n = 494)	Image-based sexual abuse (n = 335)	Nonconsensual sexting (n = 222)	Self-produced child sexual abuse images (n = 234)	Online grooming with adult perpetrator (n = 208)	Revenge pornography (n = 80)	Sextortion (n = 81)	Online commercial exploitation (n = 58)
Child age, y									
≤12	12.9 (2.3)	15.9 (2.8)	10.4 (2.3)	13.1 (3.3)	7.4 (2.2)	12.2 (4.4)	8.1 (3.7)	8.6 (3.4)	11.1 (5.1)
13-15	47.0 (3.1)	59.6 (3.6)	60.1 (4.2)	59.3 (5.1)	61.2 (5.1)	43.7 (4.8)	53.8 (8.3)	32.3 (8.6)	41.3 (8.4)
16-17	34.8 (3.0)	58.1 (3.6)	60.0 (4.3)	42.6 (5.2)	55.1 (5.5)	62.5 (4.9)	38.0 (7.8)	59.1 (8.6)	47.6 (8.4)
Perpetrator age, y									
<18	16.3 (2.3)	29.9 (3.4)	39.0 (4.0)	46.6 (5.4)	35.1 (5.3)	NA	51.7 (8.4)	31.4 (8.0)	12.8 (5.9)
18-25	8.0 (1.7)	36.2 (3.3)	31.4 (3.8)	18.5 (3.3)	26.1 (4.0)	70.8 (4.5)	14.3 (5.0)	24.5 (7.5)	16.3 (6.1)
≥26	1.4 (0.4)	14.5 (2.2)	14.0 (2.8)	8.8 (3.0)	12.0 (3.2)	26.4 (4.5)	18.6 (6.4)	3.5 (1.8)	9.7 (3.6)
Do not know or missing	74.3 (2.7)	33.6 (3.7)	24.8 (4.1)	28.7 (5.2)	31.8 (5.5)	NA	19.1 (5.8)	40.6 (8.6)	61.0 (8.0)
Perpetrator identity									
Any offline known	23.5 (2.6)	62.0 (3.8)	64.6 (4.5)	65.2 (5.4)	55.5 (5.5)	79.5 (3.5)	76.6 (6.7)	45.8 (8.5)	30.0 (7.6)
Intimate partner	6.1 (1.3)	33.5 (3.3)	39.0 (4.2)	38.2 (5.3)	37.1 (5.2)	28.5 (4.3)	50.1 (8.5)	31.6 (7.9)	17.0 (7.0)
Friend or relative	7.3 (1.8)	14.9 (2.3)	13.5 (2.8)	13.5 (3.3)	11.6 (3.3)	24.6 (4.6)	10.9 (4.3)	4.5 (1.8)	5.8 (2.6)
Acquaintance	10.1 (1.8)	23.4 (2.7)	20.3 (2.9)	16.3 (3.4)	14.9 (3.0)	34.6 (5.0)	20.2 (6.3)	9.6 (4.1)	7.2 (3.3)
Online only acquaintance	1.7 (0.6)	11.0 (2.3)	11.9 (3.0)	4.3 (2.2)	9.8 (2.8)	18.7 (3.3)	0.6 (0.4)	13.6 (7.1)	4.9 (3.4)
Do not know or missing	74.8 (2.7)	34.1 (3.7)	31.7 (4.4)	32.5 (5.3)	35.9 (5.6)	5.7 (1.9)	25.5 (6.9)	40.6 (8.6)	65.1 (7.9)

Abbreviation: NA, not applicable.

^a^ Percentages may total more than 100 because respondents may have reported more than 1 episode.

Another frequently reported type of episode (10.3% [SE, 0.8%]) was the experience of being threatened, forced, or strongly pressured to provide someone with sexual images (Table 1). The next most frequently reported type of episode (8.6% [SE, 0.7%]) was engaging in intimate sexual conversations or the sharing of sexual pictures or videos with a person 5 or more years older even if voluntary. The prevalence rates were 4.9% (SE, 0.6%) for nonconsensual image sharing, 2.0% (SE, 0.3%) for nonconsensual image taking, and 3.5% (SE, 0.6%) for being threatened with such sharing. The prevalence rates were 1.5% (SE, 0.3%) for commercial talk, 1.1% (SE, 0.2%) for commercial images, and 0.8% (SE, 0.2%) for other commercial activity.

We aggregated these specific rates of screener endorsements to represent broader concepts that have been discussed in the literature and measured in other surveys (Table 1). These aggregated categories overlap in various ways. Online child sexual abuse, a broad category, had a prevalence rate of 15.6% (SE, 1.0%) of the sample. Image-based sexual abuse, another broad concept but limited to images, had a prevalence rate of 11.0% (SE, 0.9%).

Nonconsensual sexting had prevalence rate of 7.2% (SE, 0.7%). This concept included images shared nonconsensually, taken nonconsensually, or obtained through force or pressure (if images were provided).

Self-produced child sexual abuse image (prevalence rate, 7.2% [SE, 0.7%]) included providing images to perpetrators who nonconsensually shared them, providing an image under pressure or force, and the voluntary sharing of images with adults or in commercial image transactions. This offense did not include other kinds of voluntary image sharing with a peer if no nonconsensual use occurred.

Online grooming, exclusively solicitations from and exchanges with adults, had a prevalence rate of 5.4% (SE, 0.5%). Revenge pornography (prevalence rate, 3.1% [SE, 0.5%]) covered only nonconsensual sharing or taking of images when the motive, as reported by the respondent, was specifically to harm them.

The prevalence rate for sextortion or sexual extortion, limited to someone making threats to share images already in their possession, was at 3.5% (SE, 0.6%). The 3 types of online commercial sexual exploitation (commercial talk, images, or other) aggregated to a total prevalence of 1.7% (SE, 0.3%).

Table 2 indicates that youth in the 13- to 17-year-old range comprised the large majority of targets for every episode type (81.8% for the total aggregate online child sexual abuse). Offenses against those 12 years or younger comprised less than 16% in all categories.

Table 2 also lists the perpetrator characteristics. In all categories, perpetrator age was unknown for a large percentage of episodes, increasing to a prevalence of 74.3% (SE, 2.7%) for online solicitation and 61.0% (SE, 8.0%) for commercial exploitation. Among perpetrators with known age, youthful offenders younger than 18 years made up a considerable proportion, particularly (35%-52%) for image-based sexual abuse (39% [SE, 4.0%]), nonconsensual sexting (46.6% [SE, 5.4%]), self-produced child sexual abuse images (35.1% [SE, 5.3%]), and revenge pornography (51.7% [SE, 8.4%]).

The perpetrator identity section of Table 2 also indicates that persons known offline constituted most perpetrators (55.5%-79.5%), except for 3 categories in which there were particularly large numbers of unknown perpetrators (online solicitation, 74.8% [SE, 2.7%]; sextortion, 40.6% [SE, 8.6%]; and online commercial exploitation, 65.1% [SE, 7.9%]). Few perpetrators were online-only acquaintances. The aggregate type with the largest percentage of online-only acquaintances was online grooming with an adult perpetrator, which still represented only 18.7% (SE, 3.3%) of these episodes.

Table 3 lists the rates of all categories of technology-facilitated abuse by gender. The rates for overall online child sexual exploitation and abuse and almost all of its subcategories were more than 2 times higher among female than male individuals. Transgender and gender-fluid individuals also reported substantially higher rates than cisgender male individuals for most subcategories.

**Table 3.** Prevalence Rate by Gender for Each Offense Type

Offense	Weighted prevalence rate (SE), %								
	Online solicitation (n = 732)	Online child sexual abuse (n = 494)	Image-based sexual abuse (n = 335)	Nonconsensual sexting (n = 222)	Self-produced child sexual abuse images (n = 234)	Online grooming with adult perpetrator (n = 208)	Revenge pornography (n = 80)	Sextortion (n = 81)	Online commercial exploitation (n = 58)
Gender									
Male (n = 820)	9.2 (1.3)	7.6 (1.2)	5.4 (1.0)	3.5 (0.8)	3.8 (0.9)	2.4 (0.6)	1.7 (0.6)	1.5 (0.6)	1.0 (0.3)
Female (n = 1762)	34.6 (1.8)	23.3 (1.5)	16.3 (1.4)	10.5 (1.1)	10.4 (1.1)	8.4 (0.8)	4.4 (0.8)	5.5 (1.0)	2.2 (0.4)
Transgender or gender fluid (n = 57)	48.1 (10.5)	19.7 (6.4)	15.4 (5.7)	15.1 (5.7)	11.5 (4.9)	1.8 (1.1)	4.5 (2.5)	3.9 (2.3)	7.2 (3.6)
χ^2^ test statistic	256.83	121.69	79.33	52.44	43.44	47.88	16.38	31.20	13.91
*P* value	<.001	<.001	<.001	<.001	<.001	<.001	.01	.001	.003

## Discussion

This survey study provides more nuanced information about technology-facilitated sexual abuse of juveniles than has been available in previous epidemiologic studies.^14^ These more nuanced distinctions are required because definitional categories are still in flux, and the large variety of concepts being used in research and advocacy have often not been clearly operationalized.^20^

Results indicate that rates vary considerably, depending on the categories of particular interest, ranging from 22.5% for online solicitation to 1.7% for commercial exploitation. Solicitation is a category that includes many brief episodes of unknown origin, and its frequency can inflate any aggregate to which it is added. Our understanding of the concept of sexual abuse and grooming in the literature has led us to exclude solicitations from the concept of online child sexual abuse or grooming, except when these solicitations came from an identified adult. This understanding corresponds to the convention that unwanted peer inquiries about sexual matters are not typically counted as sexual abuse in the offline context.^25^

Despite the variability of these categories, many of their features were consistent. Girls were more vulnerable than boys. Teens were the more frequent targets. Teens were also frequent perpetrators, as were young adults. Adults older than 26 years comprised only a small proportion of the offenders. The proportions of perpetrators who were in-person intimate partners, friends, and acquaintances outnumbered those who were online-only contacts. The exact proportions were complicated somewhat by a large percentage of perpetrators whose identity was unknown to or unreported by respondents. It should not, however, be presumed that unknown perpetrators were only adults or strangers, because acquaintances and other youth also have motives to hide their identities.

An important implication of these findings is that technology-facilitated abuse of children is diverse. It is not only adults or online strangers who are responsible for the problem. The problem includes peers or somewhat older young adult friends who pressure youth for sexual images or activities or who nonconsensually misuse images they have received. This abuse includes many romantic partners and offline friends.

The current study makes clear that statistics gathered in previous technology-facilitated abuse studies^26,27,28^ should not be simply interpreted as assessments of child abuse perpetrated by unknown adult groomers and molesters on the internet. The problem is exacerbated by the common use of the term *sexual abuse*, which is often presumed to imply an adult perpetrator. However, given the common dynamics of online sexual abuse in evidence here, many episodes might be better characterized as forms of intimate partner abuse, sexual harassment, and sexual bullying.^29^

The diversity of technology-facilitated abuse has important implications for prevention. Education efforts need to go beyond simply warning youth that online contacts may not be who they claim to be.^30^ Youth need to develop a set of skills that are tailored to different situations and sources of risk, including being alert to efforts at coercive control by friends and romantic partners. They need to know how to deflect and dissuade requests and pleas for sexual activities and images that are unwanted, and they need to become proficient in setting boundaries.

Considering the need for such skills, we believe there may be some benefit to integrating online safety tools into existing evidence-based programs in sex education, bullying, and dating violence prevention.^30,31^ These programs have been subject to more refinement, have a more extensive research base, and have greater staff training and experience than new, experimental, stand-alone internet safety programs.

### Strengths and Limitations

This study has a number of strengths, including a comprehensive assessment of online abuse using a multiple-question survey, detailed follow-ups, and a nationally representative sample. However, the study has some notable limitations as well. Like most contemporary surveys, the overall participation rate was low. However, the probability-based sample, efforts to reduce sample bias, and weighting strategies increase our confidence in the accuracy of our results. In addition, the episodes being analyzed in this study were retrospective accounts, some more than 10 years old. In the rapidly changing digital world, these accounts may not reflect current reality for young people. Moreover, respondents may have forgotten or misremembered details of events that occurred several years ago.

## Conclusions

The results of this national survey study indicate that a substantial proportion of young people have experienced online child sexual abuse. Professionals planning prevention and intervention strategies for online sexual abuse must understand that dynamics include diverse episodes that are often extensions of dating abuse, sexual bullying, and sexual harassment, not only events perpetrated by adult internet predators.
